# C1q/Tumor necrosis factor-related protein-3 protects macrophages against LPS-induced lipid accumulation, inflammation and phenotype transition via PPARγ and TLR4-mediated pathways

**DOI:** 10.18632/oncotarget.19657

**Published:** 2017-07-28

**Authors:** Jiale Lin, Qi Liu, Hui Zhang, Xingtao Huang, Ruoxi Zhang, Shuyuan Chen, Xuedong Wang, Bo Yu, Jingbo Hou

**Affiliations:** ^1^ Department of Cardiology, The Second Affiliated Hospital of Harbin Medical University, Harbin, China; ^2^ The Key Laboratory of Myocardial Ischemia, Harbin Medical University, Ministry of Education, Harbin, China

**Keywords:** C1q/tumor necrosis factor-related protein-3 (CTRP3), macrophage phenotype, inflammation, cholesterol efflux, atherosclerosis

## Abstract

Macrophage inflammation and foam cell formation are critical events during the initiation and development of atherosclerosis (AS). C1q/tumor necrosis factor-related protein-3 (CTRP3) is a novel adipokine with anti-inflammatory and cardioprotection properties; however, little is known regarding the influence of CTRP3 on AS. As macrophages play a key role in AS, this study investigated the effects of CTRP3 on macrophage lipid metabolism, inflammatory reactions, and phenotype transition, as well as underlying mechanisms, to reveal the relationship between CTRP3 and AS. CTRP3 reduced the number of lipid droplets, lowered cholesteryl ester (CE), total cholesterol (TC), and free cholesterol (FC) levels, reduced the CE/TC ratio, and dose-dependently inhibited TNFα, IL-6, MCP-1, MMP-9 and IL-1β release in lipopolysaccharide (LPS)-stimulated THP-1 macrophages and mouse peritoneal macrophages. Pretreatment with CTRP3 effectively increased macrophage transformation to M2 macrophages rather than M1 macrophages. Western blotting showed that the specific NF-κB pathway inhibitor ammonium pyrrolidine dithiocarbamate (PDTC) or siRNA targeting PPARγ/LXRα markedly strengthened or abolished the above-mentioned effects of CTRP3, respectively. These results show that CTRP3 inhibits TLR4-NF-κB pro-inflammatory pathways but activates the PPARγ-LXRα-ABCA1/ABCG1 cholesterol efflux pathway. Taken together, CTRP3 participates in anti-lipid accumulation, anti-inflammation and macrophage phenotype conversion via the TLR4-NF-κB and PPARγ-LXRα-ABCA1/ABCG1 pathways and, thus, may have anti-atherosclerotic properties.

## INTRODUCTION

Atherosclerosis (AS) is a chronic inflammatory disease that is closely related to abnormal lipid metabolism and maladaptive inflammatory responses, in which macrophages play pivotal roles [1]. Macrophage lipid metabolism disorders in the arterial wall lead to retention of macrophages and chronic inflammation; thus, macrophage phenotypic polarization further promotes AS progression [2]. The *in vivo* phenotype of macrophages represents a complex and dynamic state in which micro-environmental factors such as cytokines and lipid signals can polarize macrophages toward different phenotypes, which are usually categorized as classical pro-inflammatory M1 macrophages and alternative anti-inflammatory M2 macrophages [3]. M1 macrophages are activated by lipopolysaccharide (LPS) and interferon-γ, secrete pro-inflammatory cytokines such as TNF-α and IL-1β, and express the co-stimulatory molecules CD80 and CD86. In contrast, M2 macrophages are activated by IL-4 and IL-13, secrete anti-inflammatory cytokines such as IL-10, and express high levels of Arginase 1, CD163, mannose receptor (MR / CD206) and FIZZ1 [2]. In general, M1 macrophages accelerate plaque inflammation, and M2 macrophages resolve plaque inflammation. Although the mechanisms that link lipid deposition to inflammatory responses in macrophages have not yet been defined, both liver X receptors (LXRs) and peroxisome proliferator-activated receptors (PPARs) are important lipid sensors that not only regulate lipid metabolism but also exhibit anti-inflammatory properties in macrophages [4, 5]. Accumulation of cellular cholesterol activates LXR and, hence, induces expression of ATP-binding cassette subfamily A member 1 (ABCA1) and ABCG1, which facilitate the efflux of lipids and exert anti-inflammatory effects [6]. In addition, an elevated lipid content can induce pro-inflammatory signaling via Toll-like receptor (TLR)-induced NF-κB activation, resulting in increased production of pro-inflammatory cytokines and lipid intake [7–9]. By stimulating the LXRα-ABCA1/ABCG1 pathway, activated PPARγ displays anti-atherosclerotic potential [10], it also primes human monocytes toward alternative anti-inflammatory M2 macrophages [11], indicating the vital function of PPARγ in macrophages and AS.

Adipokines function in diverse physiological and pathological processes including lipid metabolism and inflammation. One adipokine, C1q/tumor necrosis factor-related protein-3 (CTRP3), is an adiponectin paralog with prominent anti-inflammatory and cardiovascular protective potential [12]. For example, it has been demonstrated that CTRP3 exhibits anti-inflammatory properties *in vitro* by inhibiting chemokine release by monocytes and adipocytes induced by lauric acid, LPS and other TLR ligands; the potential mechanism may be closely related to inhibition of LPS binding to TLR4 and suppression of NF-κB signaling [13, 14]. However, controversy still exists with regard to CTRP3’s anti-inflammatory properties *in vivo*. It has been reported that treatment of mice with CTRP3 intraperitoneally prior to LPS stimulation significantly attenuated LPS-induced cytokine levels [15], and others found that mice deficient in CTRP3 have more severe inflammatory joint pathology and higher pro-inflammatory cytokine mRNAs than wild-type mice [16]. In addition, Wolf RM et al. found that knockout of CTRP3 was insufficient to alter systemic metabolic outcomes [17], and Petersen PS et al. reported that neither CTRP3 deficiency nor overexpression in transgenic mice had an impact on IL-1β, IL-6, TNFα or MIP-2 induction at the serum protein or mRNA levels upon LPS challenge [18]. Nonetheless, the circulating CTRP3 concentration is related to cardiometabolic risk factors [19], and levels were found to be significantly lower in obese compared to lean individuals [20]. Additionally, plasma and myocardial expression of CTRP3 in mice was significantly decreased following myocardial infarction (MI), whereas CTRP3 replenishment improved mouse survival, restored cardiac function, attenuated myocardial hypertrophy, decreased the number of myofibroblasts post-MI [21, 22], and may be beneficial for attenuating vascular remodeling [23]. These findings reveal a cardioprotective capacity of CTRP3. As an adipokine, CTRP3 also functions in regulating lipid metabolism, as recombinant CTRP3 treatment regulated hepatic lipid metabolism and displayed therapeutic potential for attenuating hepatic steatosis [24]. As mentioned above, CTRP3 has beneficial effects on chronic inflammation and cardiovascular protection. Regardless, the effect of CTRP3 on AS remains largely unknown, and it is unclear whether CTRP3 can modulate lipid metabolism or phenotype polarization of macrophages. Accordingly, in this study, we explored the effects of CTRP3 on lipid deposition, inflammatory reactions and phenotypic modulation in LPS-triggered macrophages, and further investigated whether the potential mechanism is related to the TLR4-NF-κB and PPARγ-LXRα-ABCA1/ABCG1 pathways, so as to determine whether CTRP3 influences the development of AS.

## RESULTS

### CTRP3 reduced ox-LDL-induced lipid deposition in LPS-stimulated macrophages

**Figure 1:**
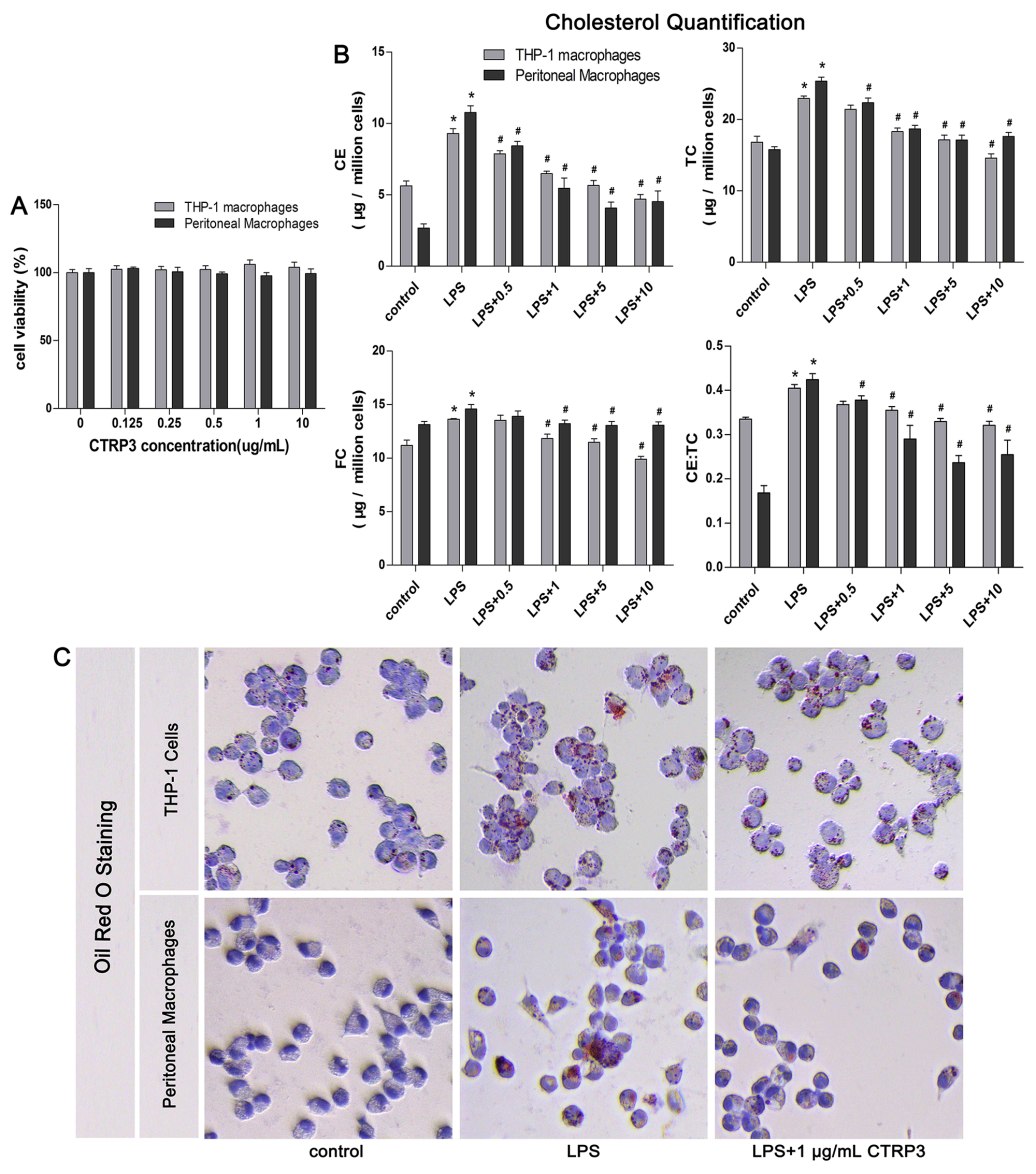
CTRP3 reduces ox-LDL-induced foam cell formation and lipid deposition in LPS-stimulated macrophages **(A)** Impacts of increasing CTRP3 concentration on the viability of THP-1 differentiated macrophages and mouse peritoneal macrophages. Cells were suspended into 1 × 10^4^ cells /100μL/well; THP-1 cells were induced to differentiate into macrophages by culture with 100 ng/mL PMA for 48 h. Mouse peritoneal macrophages were extracted and cultured as mentioned above, after which increasing doses of CTRP3 (0-10 μg/mL) were added; 24 h later, the CCK-8 assay was used to detect viability (mean ± SD). **(B-C)** Impacts of CTRP3 on LPS-triggered macrophage lipid deposition and foam cell formation in both kinds of macrophages (1 × 10^6^ cells/mL/well). The cholesterol content was detected using a cholesterol quantification kit to evaluate lipid deposition in LPS-triggered macrophages (B, mean ± SD, * : compared to the control group, P < 0.05; # : compared to the ox-LDL and LPS treatment group, P < 0.05). Oil red O staining was performed to analyze the effect of CTRP3 on foam cell formation (C, THP-1 cells: 200×, mouse peritoneal macrophages: 400×). At least three independent experiments were performed.

Macrophages can take up ox-LDL via macropinocytosis, phagocytosis and scavenger receptor-mediated uptake, forming foam cells that accelerate atherosclerotic plaques formation [2]. By measuring cell viability after incubation with various concentrations of CTRP3 (0 - 10 μg/mL) using the CCK-8 assay, we found that up to 10 μg/mL CTPR3 did not significantly decrease the viability of THP-1 macrophages and mouse peritoneal macrophages (Figure 1A). To determine whether CTRP3 can influence lipid metabolism in LPS-stimulated macrophages, THP-1 cells were differentiated into macrophages by 100 ng/mL PMA stimulation for 48 h and then incubated with 100 ng/mL LPS for 24 h prior to 100 μg/mL ox-LDL for 24 h. Mouse peritoneal macrophages were incubated with 50 ng/mL LPS for 2 h prior to 50 μg/mL oxidized LDL (ox-LDL) for 24 h, with or without CTRP3 (from 0 to 10 μg/mL) pretreatment for 30 min. Cholesterol quantification and oil red O staining were then performed as described above. The results of cholesterol quantification showed that LPS and ox-LDL stimulation elevated the cholesterol content in in both cell lines, with 1 μg/mL to 10 μg/mL CTRP3 significantly decreasing CE, TC, FC and the CE/TC ratio in LPS-stimulated macrophages (Figure 1B). Oil red O staining also showed that compared with the control group, cells treated with LPS and ox-LDL contained more red lipid droplets and displayed the morphological characteristics of foam cells. However, CTRP3 pretreatment at 1 μg/mL led to a decrease in lipid droplets (Figure 1C), suggesting that CTRP3 may restrain ox-LDL-induced foam cell formation and induce cholesterol efflux in LPS-stimulated macrophages.

### CTRP3 decreased inflammatory factors produced by LPS-triggered foam cells

**Figure 2:**
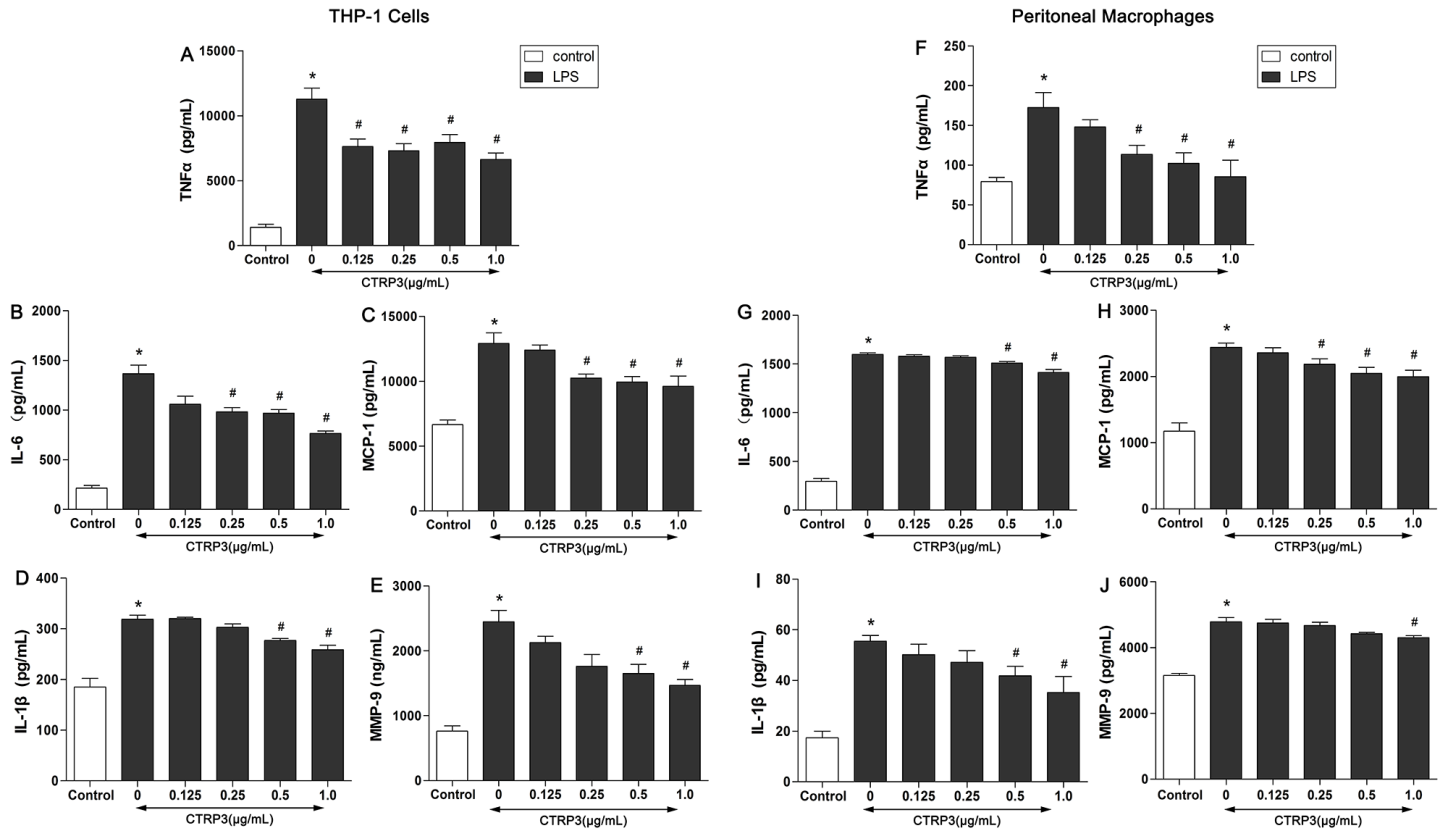
CTRP3 decreases inflammatory factors produced by LPS-triggered foam cells THP-1 cells and mouse peritoneal macrophages (1 × 10^6^ cells/mL/well) were preincubated with different concentrations of CTRP3 (0-1.0 μg/ mL) for 30 min. After LPS and ox-LDL stimulation, the supernatant protein levels of TNFα, IL-6, MCP-1, IL-1β and MMP-9 were evaluated by ELISA. At least three independent experiments were performed (mean ± SD, * : compared to the control group, P < 0.05, # : compared to the LPS and ox-LDL treatment group, P < 0.05).

The inflammatory reaction induced by TLR-4 ligands (such as LPS) is an important event in AS, and studies have identified CTRP3 as a negative regulator of inflammatory responses in monocytes and 3T3-L1 adipocytes stimulated by LPS [13, 14]. To explore whether CTRP3 has an anti-inflammatory function in LPS-triggered foam cells, THP-1 cells and mouse peritoneal macrophages were cultured as mentioned above, and the effects of CTRP3 on LPS-induced release of TNF-α, IL-6, MCP-1, IL-1β and MMP-9 were investigated by ELISA. As shown in Figure 2, LPS and ox-LDL stimulation prominently elevated supernatant TNF-α, IL-6, MCP-1, IL-1β and MMP-9 levels in both THP-1 macrophages and mouse peritoneal macrophages (P < 0.001), and the pro-inflammatory impact of LPS and ox-LDL was dose-dependently antagonized by CTRP3 at 0.125 – 1 μg/mL (P < 0.05). Moreover, a concentration of 1 μg/mL CTRP3 was effective for all five inflammatory cytokines in both cell lines. As 1 μg/mL CTRP3 significantly regulated lipid metabolism in foam cells, we chose this concentration for further experiments. According to the CCK-8 assay, CTRP3 has no cytotoxic effect on macrophages; therefore, we excluded a cytotoxic effect of CTRP3 as responsible for the observed reduction in cytokine secretion.

### CTRP3 induced transformation into M2 macrophages, as opposed to M1 macrophages

**Figure 3:**
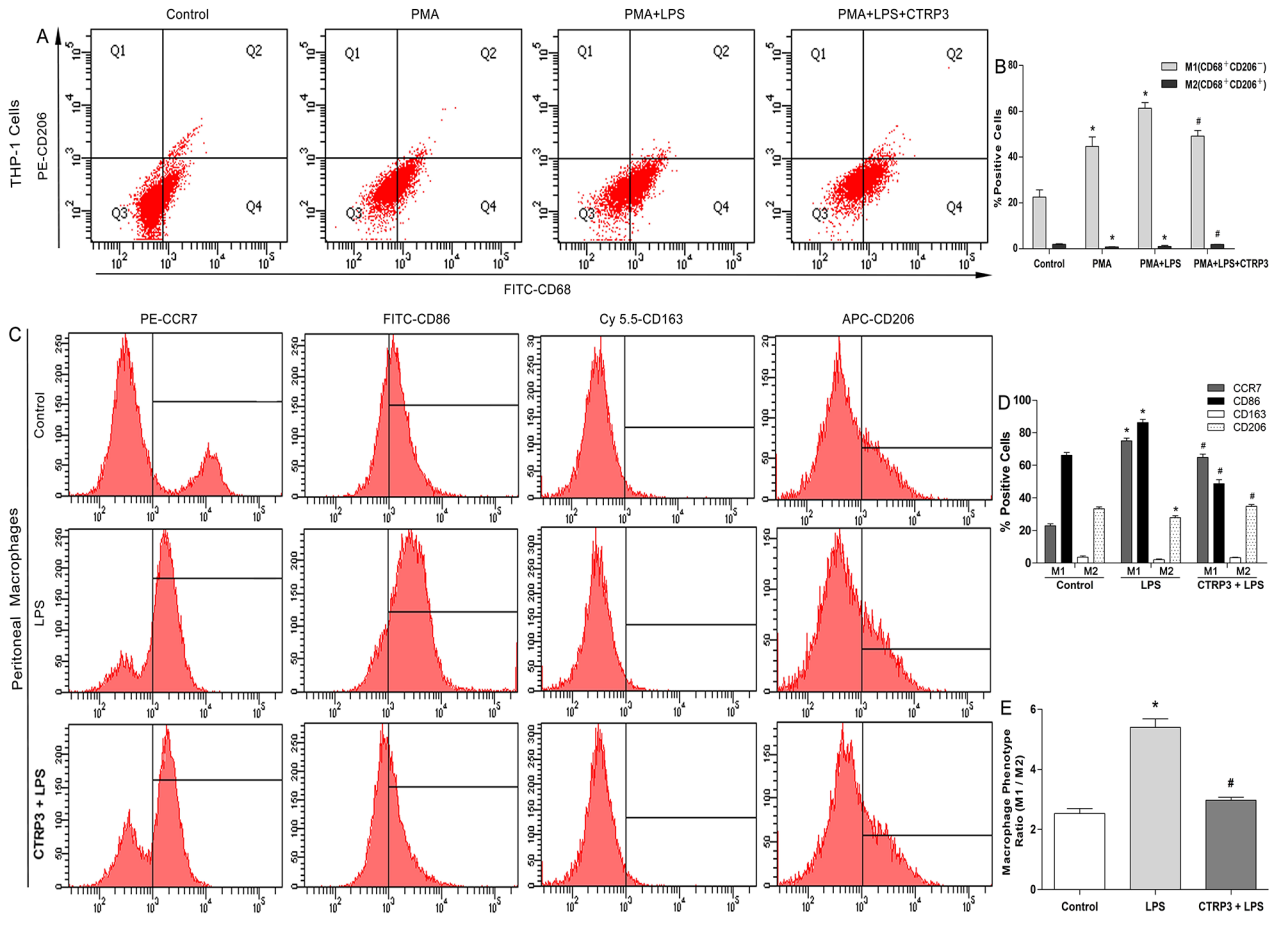
Impacts of CTRP3 on LPS-induced cell phenotype transition in macrophages Cells (1 × 10^6^ cells /mL/well) were treated, and expression of specific M1 and M2 markers on the cell surface was detected by FCM to observe macrophages polarization. For THP-1 cells, cells expressing CD68 but not CD206/MR (CD68^+^CD206^−^) were considered to be M1 macrophages; cells co-expressing CD68 and CD206 (CD68^+^CD206^+^) were considered to be M2 macrophages. For mouse peritoneal macrophages, cells expressing CCR7 and CD86 were considered to be M1 macrophages; cells expressing CD163 and CD206/MR were considered to be M2 macrophages. The macrophage phenotype ratio was calculated as (CCR7 plus CD86 expression)/(CD163 plus CD206 expression). At least three independent experiments were performed (mean ± SD, * : compared to the control group, P < 0.05, # : compared to the LPS but without CTRP3 treatment group, P < 0.05).

**Figure 4:**
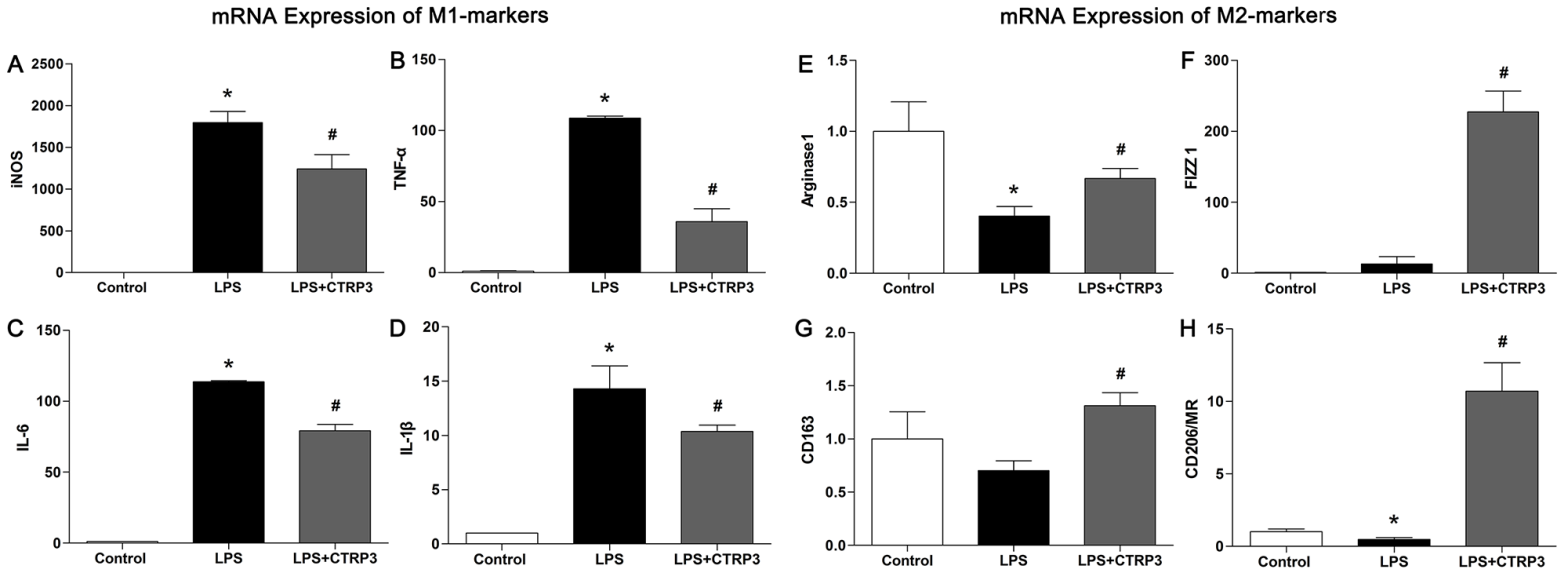
CTRP3 alters mRNA expression of M1 and M2 markers in macrophages PMA-induced THP-1 macrophages (1 × 10^6^ cells/mL/well) were divided into three groups: the control group was treated with PBS for 24 h; the LPS group was treated with 100 ng/mL LPS for 24 h; the LPS+CTRP3 group was treated with 1 μg/mL CTRP3 for 30 min before LPS stimulation for 24 h. mRNA expression of specific M1 marker and M2 markers was assessed by qRT-PCR. Relative mRNA concentrations were calculated using the ΔΔCT method, and the expression level was normalized to that of endogenous β-actin. At least three independent experiments were performed (mean ± SD, * : compared to the control group, P < 0.05, # : compared to the LPS group, P < 0.05).

Macrophages are heterogeneous cells, and M1 and M2 macrophages are the primary cell components of atherosclerotic lesions, contributing to foam cell formation and inflammatory factor secretion [25]. M1 macrophages are activated by LPS or other TLR ligands, and the activated cells express NF-κB and secrete pro-inflammatory cytokines such as TNFα and IL-6; Th2 cytokines (e.g., IL-4 and IL-13) induce alternative M2 macrophage activation, and activated M2 cells show elevated expression of PPARγ and secrete anti-inflammatory cytokines such as IL-10 [11, 26]. Under specific circumstances, macrophages can switch from the M1 to M2 state and vice versa [27]. Because we demonstrated that CTRP3 has an anti-inflammation function when triggered by LPS, we further sought to clarify the role of CTRP3 in LPS-stimulated macrophage phenotype polarization. THP-1 cells were treated as follows: PBS (72 h); 100 ng/mL PMA (72 h); 100 ng/mL PMA prior to 100 ng/mL LPS (PMA 48 h + LPS 24 h); or 100 ng/mL PMA prior to 1 μg/mL CTRP3 and 100 ng/mL LPS (PMA 48 h + CTRP3 30 min + LPS 24 h). Mouse peritoneal macrophages were treated as follows: PBS (24 h); 50 ng/mL LPS (24 h); or 1 μg/mL CTRP3 for 30 min prior to 50 ng/mL LPS (24 h). FCM was performed to analyze macrophage phenotype. As shown in Figure 3, PMA alone or combined with LPS stimulation elevated the macrophage activation and reduced M2 macrophage activation of THP-1 cells. Pretreatment with CTRP3 significantly inhibited the effects, reducing the conversion to M1 macrophages and increasing that to M2 macrophages. In mouse peritoneal macrophages, LPS stimulation also enhanced M1 macrophage activation and reduced M2 macrophage activation; pretreatment with CTRP3 also significantly antagonized the effect of LPS, reducing the M1/M2 ratio. To further verify the function of CTRP3 in modulating macrophage phenotype transition, we assessed mRNA expression of certain M1 markers (iNOS, TNF-α, IL-6, IL-1β) and M2 markers (Arginase1, FIZZ1, CD163, CD206/MR) in THP-1 macrophages. As shown in Figure 4, LPS stimulation of THP-1 macrophages significantly elevated mRNA expression of M1 markers iNOS, TNF-α, IL-6, and IL-1β and decreased the level of M2 markers Arginase1 and CD206/MR. Pretreatment of CTRP3 significantly antagonized the effect of LPS, reducing the levels of iNOS, TNF-α, IL-6, and IL-1β and increasing those of Arginase1, FIZZ1, CD163 and CD206/MR. These results are consistent with the FCM results, suggesting that CTRP3 may play a role in the regulation of macrophage differentiation.

### CTRP3 suppressed TLR4-NF-κB signaling and activated the PPARγ-LXRα-ABCA1/ABCG1 pathway in macrophages

**Figure 5:**
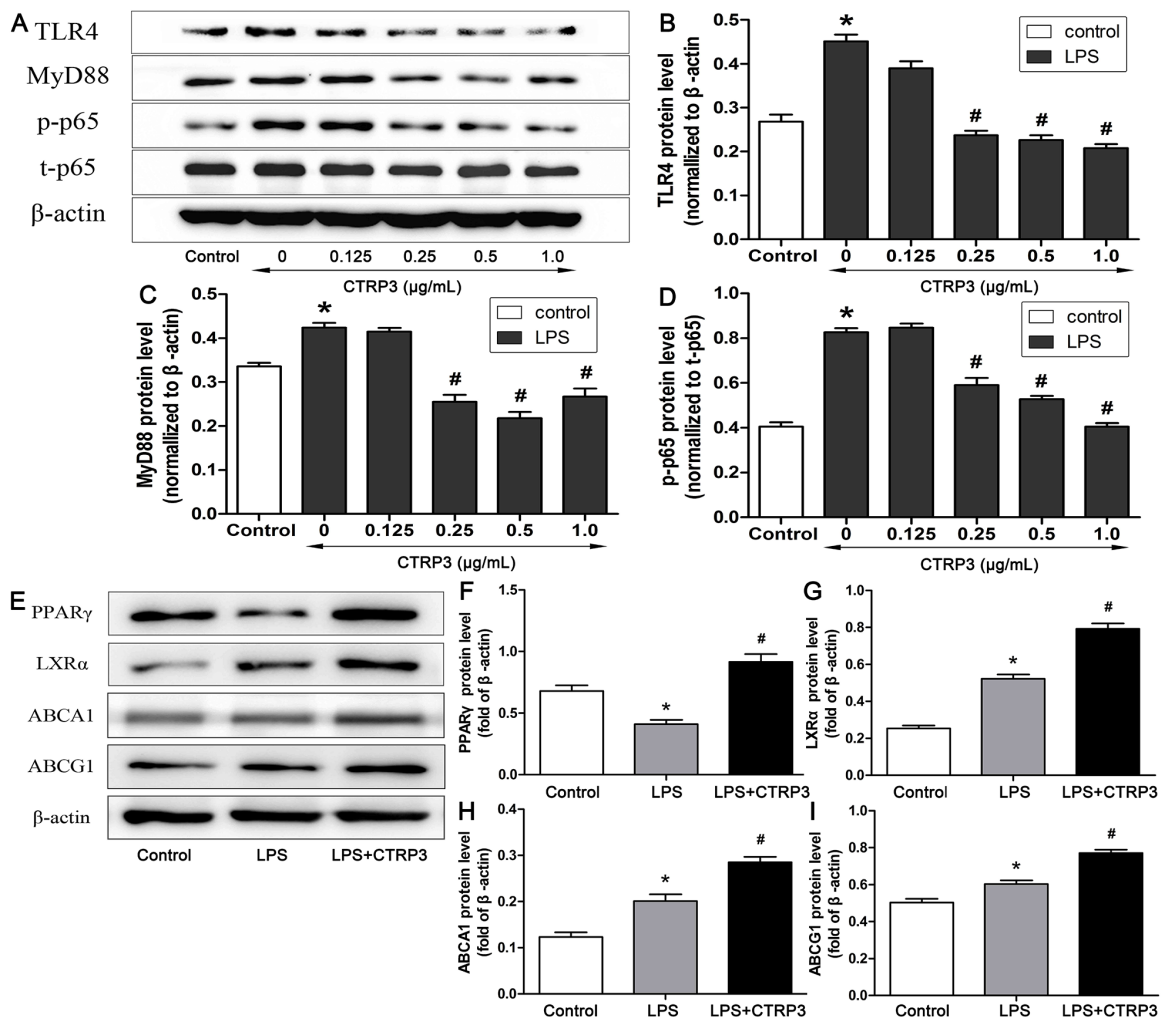
Impacts of CTRP3 on levels of TLR4-NF-κB and PPARγ-LXRα-ABCA1/ABCG1 pathway proteins in THP-1 macrophages Different concentrations of CTRP3 were added before cells (1 × 10^6^ cells/mL/well) were exposed to LPS and ox-LDL, and protein expression of TLR4, MyD88, p-NF-κB p65 and NF-κB p65 was assessed by WB **(A-D)**. Protein expression of PPARγ, LXRα, ABCA1 and ABCG1 was assessed by WB **(E-I)** (mean ± SD, * : compared to the control group, P < 0.05, # : compared to the LPS and ox-LDL treatment group, P < 0.05).

TLR4-NF-κB signaling has an important function in regulating macrophage inflammation during the process of AS [28], and the LXRα-ABCA1/ABCG1 pathway is vital to macrophage cholesterol efflux [6, 29, 30]; moreover, reports have shown that PPARγ can activate LXRα to increase downstream ABCA1/ABCG1 expression [10, 31, 32]. Therefore, to clarify the potential mechanisms involved in the inhibitory effects of CTRP3 on the inflammatory reaction and lipid deposition in macrophages, THP-1 cell expression of specific proteins related to the TLR4-NF-κB, PPARγ and LXRα-ABCA1/ABCG1 pathways were investigated. Based on WB, CTRP3 dose-dependently reduced TLR4 and downstream MyD88 protein expression as well as NF-κB p65 phosphorylation, and the effects were significant at the dose of 0.25 μg/mL (Figure 5). Conversely, 1 μg/mL CTRP3 enhanced PPARγ and LXRα protein expression, with that of ABCA1 and ABCG1 also being increased (Figure 5), indicating that the protective function of CTRP3 in macrophages may related to these pathways.

### Suppression of the TLR4-NF-κB pathway and activation of the PPARγ-LXRα-ABCA1/ABCG1 pathway are responsible for the inhibitory effect of CTRP3 on the inflammatory response and lipid accumulation

**Figure 6:**
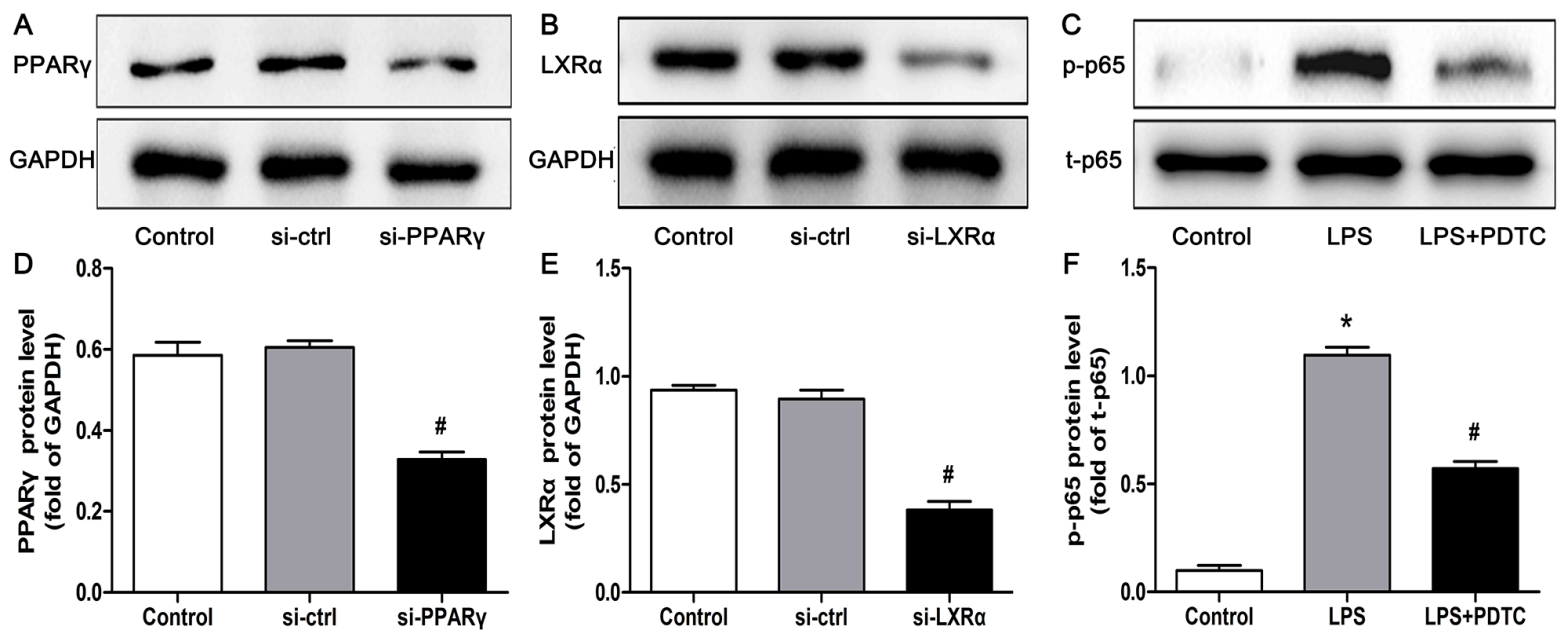
Effectiveness of si-ctrl, si-PPARγ, si-LXRα and PDTC, as verified by WB THP-1 macrophages (1 × 10^6^ cells/mL/well) were treated as mentioned above, and the protein levels of PPARγ, LXRα, p-p65 and t-p65 were detected by WB (mean ± SD, * : compared to the control group, P < 0.05, # : compared to the si-ctrl or LPS treatment group, P < 0.05).

**Figure 7:**
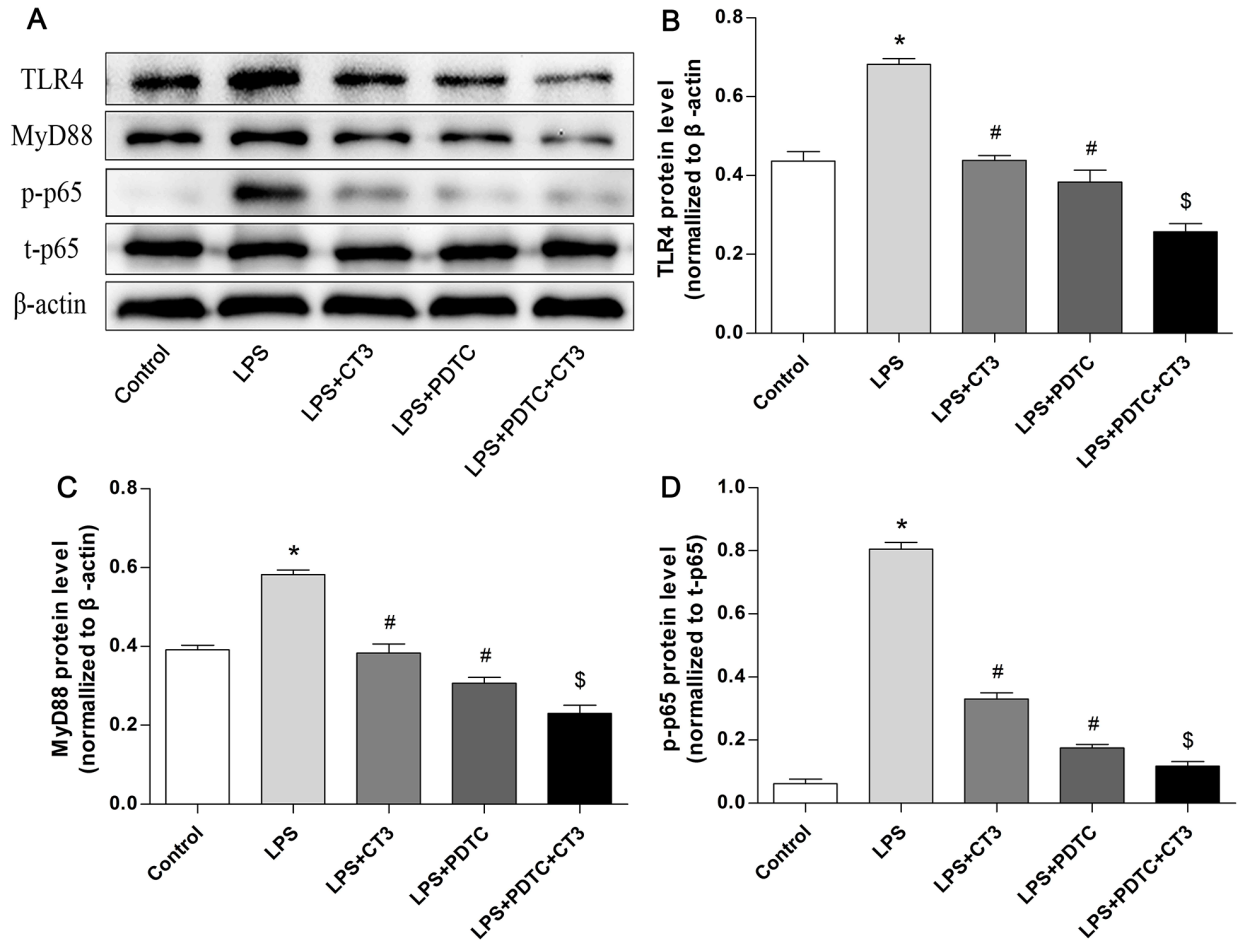
CTRP3 has an inhibitory effect on the TLR4-NF-κB pathway similar to that of the pathway inhibitor PDTC in macrophages PMA-induced THP-1 macrophages (1 × 10^6^ cells/mL/well) were divided into five groups:the control group was treated with PBS; the LPS group was treated with 100 ng/mL LPS for 24 h prior to 100 μg/mL ox-LDL for 24 h; the LPS+CTRP3 (CT3) group was pretreated with 1 μg/mL CTRP3 for 30 min before LPS and ox-LDL stimulation; the LPS+PDTC group was pretreated with 100 μM PDTC for 1 h and then stimulated with LPS and ox-LDL; the LPS+PDTC+CT3 group was treated with 100 μM PDTC for 1 h and then 1 μg/mL CTRP3 for 30 min before LPS and ox-LDL stimulation. Expression of TLR4, MyD88, NF-κB p-p65, t-p65 and β-actin was detected by WB (mean ± SD, * : compared to the control group, P < 0.05, # : compared to the LPS group, P < 0.05, $ : compared to the LPS+CT3 group, P < 0.05).

**Figure 8:**
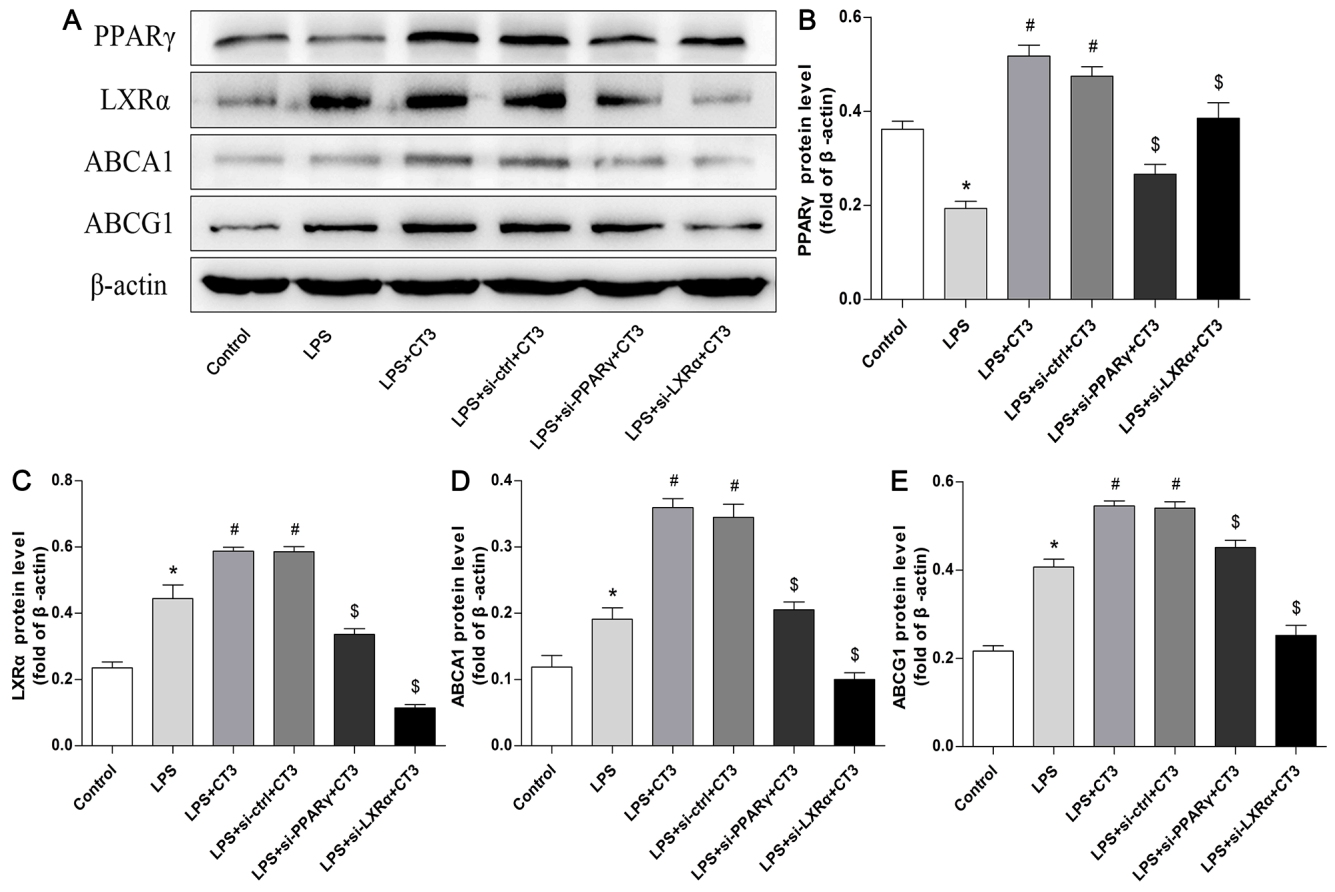
Transfection of si-PPARγ and si-LXRα reduces the function of the LXRα-ABCA1/ABCG1 cholesterol efflux pathway in macrophages PMA-induced THP-1 macrophages (1 × 10^6^ cells/mL/well) were divided into six groups: the first three group were treated as mentioned above, and the other three groups were transfected with si-ctrl, si-PPARγ or si-LXRα for 48 h, and then treated with 1 μg/mL CTRP3 for 30 min, and finally stimulated with LPS and ox-LDL. Expression of PPARγ, LXRα, ABCA1, ABCG1 and β-actin was detected by WB (mean ± SD, * : compared to the control group, P < 0.05, # : compared to the LPS group, P < 0.05, $ : compared to the LPS+CT3 group, P < 0.05).

**Figure 9:**
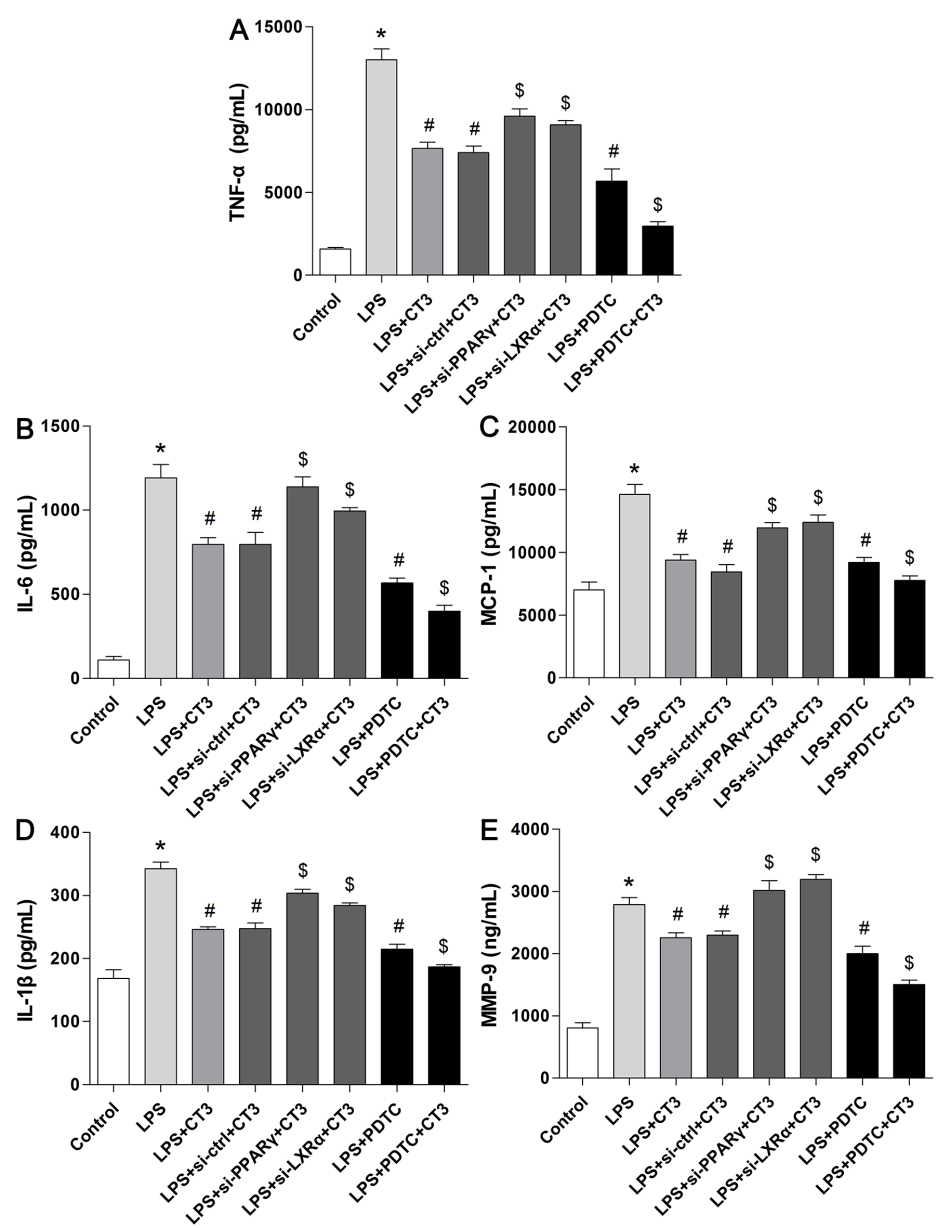
Suppression of the TLR4-NF-κB pathway and activation of the PPARγ-LXRα-ABCA1/ABCG1 pathway are responsible for the inhibitory effect of CTRP3 on the inflammatory response Cells (1 × 10^6^ cells/mL/well) were treated as mentioned above, and the supernatant protein levels of TNFα, IL-6, MCP-1, IL-1β and MMP-9 were assessed by ELISA. At least three independent experiments were performed (mean ± SD, * : compared to the control group, P < 0.05, # : compared to the LPS group, P < 0.05, $ : compared to the LPS+CT3 group, P < 0.05).

**Figure 10:**
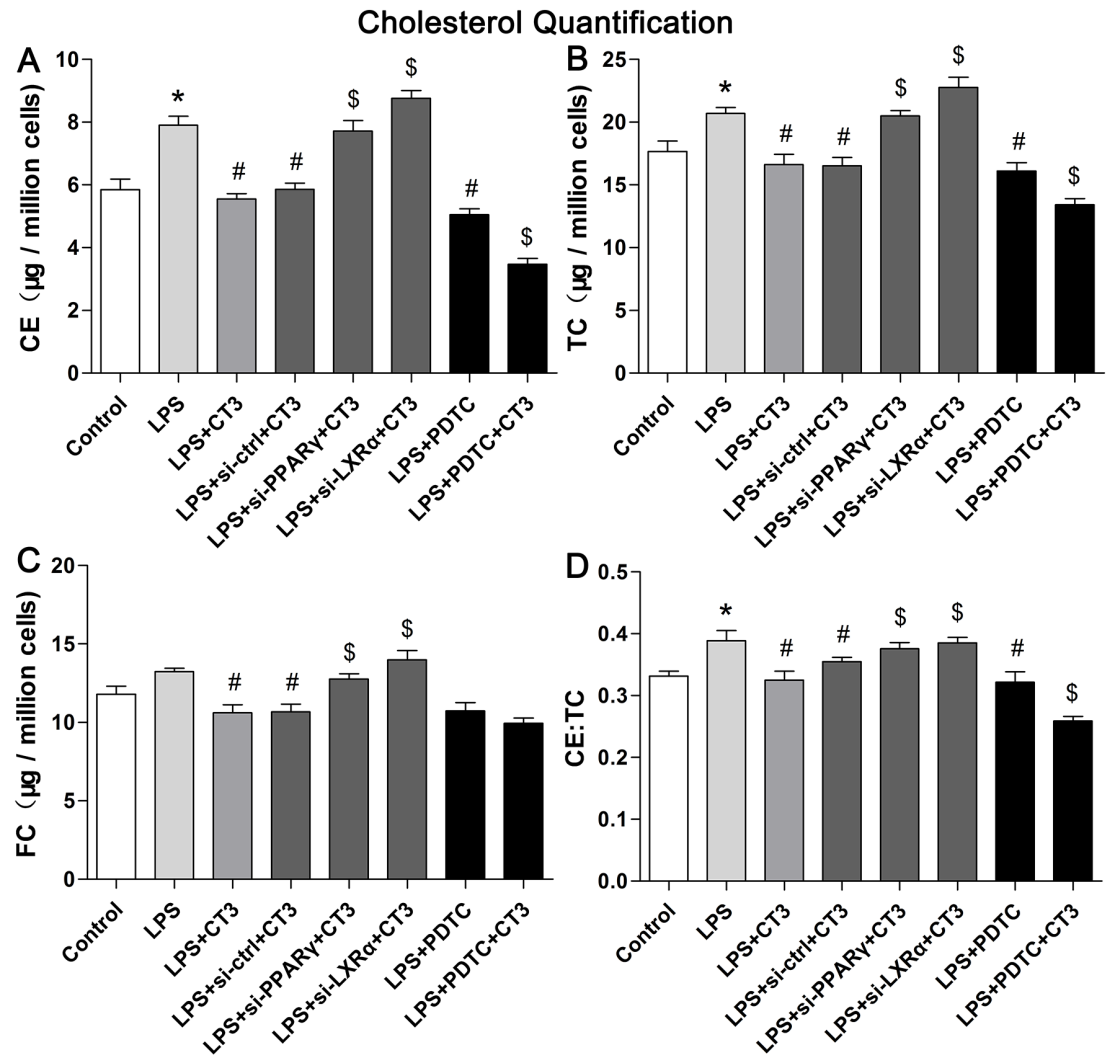
Suppression of the TLR4-NF-κB pathway and activation of the PPARγ-LXRα-ABCA1/ABCG1 pathway are responsible for the inhibitory effect of CTRP3 on lipid accumulation in macrophages Cells (1 × 10^6^ cells/mL/well) were treated as mentioned above, and the levels of CE, TC, and FC, and the CE/TC ratio were evaluated using a cholesterol quantification kit. At least three independent experiments were performed (mean ± SD, * : compared to the control group, P < 0.05, # : compared to the LPS group, P < 0.05, $ : compared to the LPS+CT3 group, P < 0.05).

**Figure 11:**
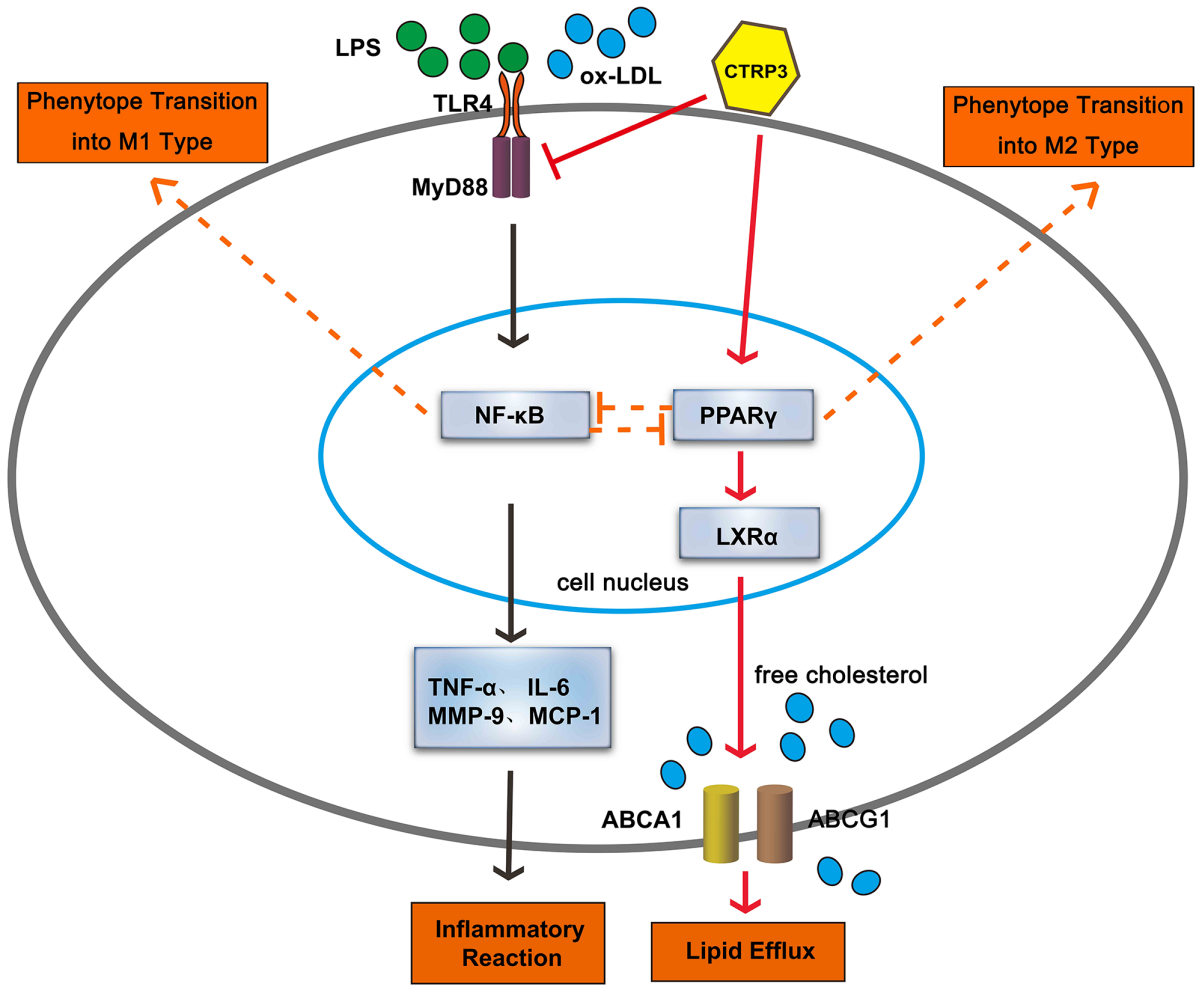
Schematic summary of the proposed mechanism by which CTRP3 modulate the function of macrophages Arrows drawn with a full line show the proposed mechanism verified in our research, and arrows drawn with a dotted line show the proposed mechanism summarized by previous research.

To verify whether CTRP3 modulates macrophage function through the TLR4-NF-κB and PPARγ-LXRα-ABCA1/ABCG1 pathways, we further treated THP-1 macrophages with the specific NF-κB pathway inhibitor PDTC and siRNAs targeting PPARγ and LXRα and evaluated production of relevant inflammatory cytokines and the cholesterol content. To verify the effect of targeted knockdown by siRNAs, we chose three pairs of siRNA as well as control siRNA and evaluated their efficiency by RT-PCR; we ultimately chose the most effective for use. Data and graphs of the efficiency of these siRNAs are shown in the Supplementary Figure 1. The efficiency of PDTC and siRNA in inhibiting particular pathways was assessed and verified by WB. PDTC strongly inhibited the level of p-NF-κB p65, and transfection of si-PPARγ and si-LXRα significantly reduced the level of PPARγ and LXRα, respectively; in contrast, si-ctrl had no significant effect on cells compared to the control group (Figure 6). PDTC significantly suppressed TLR4, MyD88 and p-NF-κB p65 expression, and the inhibitory effects were further enhanced when CTRP3 was added followed by PDTC (Figure 7). As shown in Figure 8, si-PPARγ transfection not only decreased expression of PPARγ but also reduced that of LXRα, ABCA1 and ABCG1; transfection of si-LXRα reduced LXRα, ABCA1 and ABCG1 expression, consistent with previous research [6]. Further ELISA analysis showed that PDTC combined with CTRP3 had a stronger inhibitory effect than either alone and that transfection of si-PPARγ and si-LXRα significantly abolished the effect of CTRP3 in reducing inflammatory cytokine (TNF-α, IL-6, MCP-1, IL-1β, MMP-9) production (Figure 9). The results of cholesterol quantification (Figure 10) were similar to the ELISA results, with CE, TC, and FC levels and the CE/TC ratio being further decreased by the combination of CTRP3 and PDTC, which on the contrary being restored by si-PPARγ and si-LXRα. These findings illustrate that suppression of the TLR4-NF-κB pathway as well as activation of the PPARγ-LXRα-ABCA1/ABCG1 pathway are at least partly responsible for the inhibitory effect of CTRP3 on the inflammatory response and lipid accumulation in macrophages. Schematic summary of the proposed mechanism by which CTRP3 modulate the function of macrophages is shown by Figure 11.

## DISCUSSION

Monocytes are precursors of macrophages, which are prominent cells in AS that respond to lipid accumulation and chronic inflammation. The balance of macrophages in atherosclerotic plaques is dynamic and is central to the pathophysiology of AS, whereby changes in macrophage number, inflammatory phenotype or lipid load can influence plaque fate [2]. The mechanism linking macrophage lipid metabolism to the inflammatory response is complex and remains unclear, though the TLR4-NF-κB pathway and PPARγ play important roles in these processes and are closely related to the classical LXRα-ABCA1/ABCG1 cholesterol efflux pathway [30–35]. For example, activation of TLR signaling leads to reduced cholesterol efflux, which in turn leads to cholesterol accumulation and aggravated inflammatory responses; in contrast, induction of ABCA1/ABCG1 expression by LXRs promotes cholesterol efflux and suppresses TLR-mediated inflammatory responses [7, 9]. Among numerous factors that can influence macrophage function, adipocytokines are newly discovered to function in diverse physiological and pathological processes. CTRP3 belongs to the adipokine family of adiponectin paralogs with a C-terminal complement factor C1q globular (gC1q) domain [12]. CTRP3 has been found to possess prominent anti-inflammatory and cardiovascular protection potential [36], but its roles in AS, macrophage cholesterol homeostasis and phenotype polarization are poorly understood, and the underlying mechanisms for its biological function have yet to be revealed. Therefore, we aimed to investigate the effect of CTRP3 on macrophage lipid metabolism, inflammation and phenotype transition *in vitro* to explore whether CTRP3 can influence AS by regulating macrophage function.

Our results show that treatment with human recombinant CTRP3 significantly decreased the levels of CE, TC, and FC as well as the CE/TC ratio and the number of lipid droplet in both THP-1 macrophages and mouse peritoneal macrophages. This is the first study to assess the role of CTRP3 in cholesterol homeostasis in macrophages, and our results further reveal the lipid-regulatory function of CTRP3. Data based on ELISA show that CTRP3 significantly decreased levels of pro-inflammatory factors such as TNFα, IL-6, MCP-1, IL-1β and MMP-9 produced by LPS-triggered foam cells, which was consistent with the previous research [13, 14]. Although still controversial, there is research providing evidence for the anti-inflammatory properties of CTRP3 *in vivo* [15, 16], and our data add evidence for this anti-inflammatory effect. Nonetheless, additional study is needed to further verify the functions of CTRP3. Moreover, we are the first to demonstrate that CTRP3 pretreatment of THP-1 macrophages and mouse peritoneal macrophages significantly reduces LPS-induced M1 macrophage polarization while elevating the ratio of cells transforming into M2 macrophages. These observations further verify the powerful regulatory function of CTRP3 in macrophages. Because the TLR4-NF-κB and PPARγ-LXRα-ABCA1/ABCG1 pathways are closely related to the macrophage functions mentioned above, we further identified the specific signaling molecules involved in the effect of CTRP3 on THP-1 macrophages. CTRP3 caused significant inhibition of the TLR4-NF-κB pathway yet stimulated the PPARγ-LXRα-ABCA1/ABCG1 pathway, and its modulation of these two pathways were both responsible for its anti-inflammatory and lipid-regulatory effects. The impact of PPARγ on macrophage ABCA1 expression and ABCA1-mediated cholesterol efflux is controversial: some researchers showed that high expression of PPARγ does not increase ABCA1 expression but only activates ABCG1 expression [37, 38], whereas others indicated that PPARγ activates the LXRα-ABCA1/ABCG1 pathway to induce lipid efflux [10, 31, 32, 39, 40]. Our results tend to agree with the latter and also add further evidence. Regardless, activation of PPARγ is reported to promote macrophage transition into M2 macrophages with anti-inflammatory properties rather than M1 macrophages [11], which was to a certain degree is in agreement with our experimental results.

Anti-inflammation has long been considered a means of restraining the process of AS, and more recently, the inflammatory phenotype of macrophages in AS has attracted close attention. Macrophages comprise a heterogeneous group of cells, among which classically activated M1 macrophages and alternatively activated M2 macrophages have been extensively researched. Both M1 and M2 macrophages are present in human atherosclerotic plaques, but their distribution in lesions differ widely: M2 macrophages are localized to more stable locations and are inversely related to disease progression, whereas M1 macrophages are strongly expressed in symptomatic plaques with a higher lipid content [41, 42]. Several observations to date have indicated that the progress of AS could be antagonized by constraining inflammatory M1 macrophage activation or promoting macrophage differentiation into the anti-inflammatory M2 phenotype [43, 44]. It has also been reported that ABCA1 and ABCG1 deficiency in macrophages increases inflammation and accelerates atherosclerosis in mice [45, 46]. Moreover, impairment in ABCA1-driven cholesterol efflux is associated with increased arterial-wall thickness and atherosclerosis in humans [47, 48], indicating the important role of cholesterol efflux in the process of AS. Our data demonstrate that CTRP3 possesses anti-inflammatory properties, promoting macrophage differentiation into the M2 phenotype, reducing the polarization of M1 macrophages, and inducing cholesterol efflux in macrophages, these effects possibly occur through the TLR4-NF-κB and PPARγ-LXRα-ABCA1/ABCG1 pathways. Therefore, this is the first report to demonstrate the great potential of CTRP3 in anti-atherosclerosis, though additional research is needed to further reveal the relationship between CTRP3 and AS. Our results highlight the activity of CTRP3 in modulating macrophages and reveal the underlying mechanisms. The findings suggest that CTRP3 may be particularly effective in the prevention and treatment of AS.

In conclusion, our study provides evidence that CTRP3 reduces macrophage lipid accumulation and inflammatory responses, induces macrophage cholesterol efflux and promotes transformation of macrophages toward the M2 phenotype rather than the M1 phenotype. These effects possibly occur through the TLR4-NF-κB and PPARγ-LXRα-ABCA1/ABCG1 pathways. Thus, CTRP3 may possess anti-atherosclerotic properties.

## MATERIALS AND METHODS

### Materials

RPMI (Roswell Park Memorial Institute) medium was obtained from HyClone-Thermo Fisher Scientific (Waltham, MA, USA) and fetal bovine serum (FBS) from ScienCell (Carlsbad, CA, USA). Human recombinant CTRP3 protein obtained using an *in vitro* wheat germ expression system was purchased from Abnova (Taipei, Taiwan). Cholesterol Quantitation Kit, Oil Red O solution, LPS (*Escherichia coli* serotype 055:B5), phorbol-12-myristate-13-acetate (PMA) and pyrrolidine dithiocarbamate (PDTC) were purchased from Sigma-Aldrich (St. Louis, USA). Human oxidized low-density lipoprotein (ox-LDL) was purchased from Yiyuan Biotechnologies (Guangzhou, China). All transfection reagents, siRNAs targeting human PPARγ (si-PPARγ), LXRα (si-LXRα), and GAPDH (si-GAPDH) and negative control siRNA (si-ctrl) were purchased from RiboBio Company (Guangzhou, China). Cell Counting Kit-8 (CCK-8) was obtained from Dojindo (Kumamoto, Japan). All enzyme-linked immunosorbent assay (ELISA)-based detection systems were purchased from R&D Systems (Minneapolis, USA). Fluorescein isothiocyanate (FITC)-conjugated mouse anti-human CD68, phycoerythrin (PE)-conjugated mouse anti-human CD206 (macrophage mannose receptor, MR), FITC-conjugated rabbit anti-mouse CD86 and PE-conjugated rabbit anti-mouse CCR7 were obtained from BD Biosciences (Palo Alto, CA, USA). Cy 5.5-conjugated rabbit anti-mouse CD163 was purchased from Bioss Company (Beijing, China). Mouse MR/CD206 allophycocyanin (APC)-conjugated antibody was purchased from R&D Systems (Minneapolis, USA). TRIzol was purchased from Life Technologies (Carlsbad, CA, USA), and all other quantitative reverse transcription-polymerase chain reaction (qRT-PCR)-related reagents were obtained from HaiGene Company (Harbin, China). Primary antibodies against NF-κB p65, phospho (p)-NF-κB p65, β-actin, and GAPDH and horseradish peroxidase (HRP)-conjugated secondary antibodies were obtained from Cell Signaling Technology (Beverly, MA, USA). Primary antibodies against TLR4, MyD88, PPARγ, LXRα, ABCA1 and ABCG1 were obtained from Abcam (Cambridge, MA, USA). The enhanced chemiluminescence (ECL) western blotting detection kit was purchased from Beyotime (Beijing, China).

### Cell culture

THP-1 cells were obtained from American Type Culture Collection (ATCC, Manassas, VA, USA) and cultured in RPMI 1640 medium supplemented with 10% FBS at 37°C in humidified air containing 5% CO_2_. To induce differentiation into macrophages, cells (an initial density of 1 × 10^6^ cells/mL) at P5 were cultured in the presence of 100 ng/mL PMA for 48 h. Peritoneal macrophages were obtained from male C57BL6 mice by flushing the peritoneal cavity with 5 mL of phosphate-buffered saline (PBS). The cell suspension was centrifuged, after which the cells were washed once with RPMI 1640 medium and then suspended in RPMI 1640 medium with 10% FBS and 100 IU/mL penicillin and 100 IU/mL streptomycin. After incubation for 2 h, nonadherent cells were removed, and the remaining cells were cultured for follow-up experiments. To induce foam cell formation and the inflammatory response, PMA-induced THP-1 macrophages were incubated with 100 ng/mL LPS for 24 h prior to 100 μg/mL ox-LDL for 24 h; mouse peritoneal macrophages were incubated with 50 ng/mL LPS for 2 h prior to 50 μg/mL ox-LDL for 24 h, with or without 30 min of pretreatment with different concentrations of CTRP3 (from 0 to 10 μg/mL). To explore how certain pathways are influenced by CTRP3, the specific NF-κB pathway inhibitor PDTC (100 μM) was added 1 h before CTRP3; cells were transfected with target or control siRNA (100 nmol) at 48 h before CTRP3 treatment. The cultured medium and cells were then collected for detection.

### Cell viability assay

To assess cell viability, 1 × 10^4^ macrophages were seeded in 96-well plates in complete medium and treated with different concentrations of CTRP3 (0, 0.125, 0.25, 0.5, 1, 10 μg/mL) for 24 h; 10 μL CCK-8 reagent was then added to each well according to the manufacturer’s instructions (Dojindo, Kumamoto, Japan). After incubation at 37°C for 4 h, the absorbance of each sample at 450 nm was measured using a Tecan Infinite M200 microplate reader (LabX, Austria). All experiments were performed in triplicate and repeated independently three times.

### Transfection

Prior to transfection, specific siRNA (100 nM) was incubated with 6 μL riboFECT™ CP Reagent in 60 μL PBS-diluted riboFECT™ CP Buffer for 15 min at room temperature; the transfection mixture was then added to the cell medium for 48 h. After additional treatment, cells transfected with si-ctrl or targeting siRNA were collected and used for cholesterol quantitation assays and western blotting. Inflammatory cytokines in the supernatants were measured by ELISA.

### Cholesterol concentration assay

Cultured macrophages (1 × 10^6^ cells/mL) were collected using a cell scraper. After centrifugation, cells were extracted with 200 μL chloroform: isopropanol: IGEPAL CA-630 (7: 11: 0.1) in a microhomogenizer and centrifuged at 13000 × g for 10 min to remove the insoluble material. The organic phase was transferred to a new tube and air dried at 50°C for 30 min to remove the chloroform and subjected to a vacuum for 30 min to remove the residue organic solvent. The dried lipids were dissolved in 200 mL Cholesterol Assay Buffer and vortexed. Total cholesterol (TC) and free cholesterol (FC) were detected according to the specifications of the reaction mixtures indicated by the manufacturer; the reaction was incubated at 37°C for 60 min while protected from light. Finally, the absorbance of different reactions was measured at 570 nm using a microplate reader. Concentrations of TC and FC were calculated using the sample volumes added to the wells (μL) and by dividing the amount of cholesterol in the sample according to the standard curve (μg). Cholesteryl ester (CE) was determined by subtracting FC from TC.

### Oil red O staining

After removing the supernatant, cells were washed twice with PBS, fixed for 10 min with 4% formaldehyde, and washed; 60% isopropanol was added, and the samples were dried. Oil red O solution prepared in advance was added for 15 min. The cells were washed twice with PBS and observed under an inverted microscope. Cells of each experimental group were examined within a microscopic field (THP-1 cells: *200 magnification, mouse peritoneal macrophages: *400 magnification).

### ELISA

THP-1 macrophages and mouse peritoneal macrophages were resuspended at 1 × 10^6^ cells/mL and incubated in six-well plates for the times indicated. The supernatants were collected, centrifuged, and frozen at -20°C or directly used for TNF-α, IL-6, MCP-1, IL-1β and MMP-9 protein determinations. ELISAs were performed as recommended (R&D Systems, Minneapolis, USA).

### Flow cytometry (FCM)

To analyze macrophage polarization, cells were collected and washed twice with PBS; fixation and permeabilization solution were added, and the cells were incubated for 20 min at 4°C and then washed twice with wash solution. THP-1 macrophages were incubated at 4°C in wash solution with FITC-conjugated mouse anti-human CD68 and PE-conjugated mouse anti-human CD206. Mouse peritoneal macrophages were incubated in wash solution with FITC-conjugated rabbit anti-mouse CD86, PE-conjugated rabbit anti-mouse CCR7, Cy 5.5-conjugated rabbit anti-mouse CD163 and mouse MR/CD206 APC-conjugated antibodies at 4°C. Finally, the cells were washed twice with wash solution and fixed in 4% paraformaldehyde before FCM analysis. Surface expression of specific markers was determined using an Epics Altra flow cytometer. All reagents, except antibodies, were obtained from BD Biosciences (Palo Alto, CA, USA).

For THP-1 cells, cells expressing CD68 but not CD206/MR (CD68^+^CD206^−^) were considered to be M1 macrophages, whereas cells co-expressing CD68 and CD206 (CD68^+^CD206^+^) were considered to be M2 macrophages. Regarding mouse peritoneal macrophages, cells expressing CCR7 and CD86 were considered to be M1 macrophages, and those expressing CD163 and CD206/MR were considered to be M2 macrophages. The ratio of macrophage phenotype was calculated as (CCR7 plus CD86 expression)/(CD163 plus CD206 expression) [49, 50].

### Quantitative RT-PCR

After treatment, total RNA was extracted from macrophages using TRIzol reagent and quantified using a Nanodrop ND5000 spectrophotometer. Conversion of mRNA into cDNA was performed using Golden 1st cDNA Synthesis Kit. The primer sequences used are listed in Supplementary Table 1. qRT-PCR was accomplished using a Bio-Rad Min-Opticon2 quantitative PCR system with a SYBR Green Fluorescene qPCR kit. The following optimized conditions were used: 95°C for 15 min, 95°C for 10 s and 40 cycles at 60°C for 30 s. Relative mRNA concentrations were calculated using the ΔΔCT method, and the expression level was normalized to that of endogenous β-actin or GAPDH.

### Western blotting (WB)

After treatment, cells were harvested and lysed with RIPA lysis buffer. Protein concentrations were measured using a BCA Protein Assay Kit. Equal amounts of protein from each sample were separated by 10% sodium dodecyl sulfate-polyacrylamide gel electrophoresis (SDS-PAGE) and transferred to polyvinylidene fluoride (PVDF) membranes (Millipore, Billerica, MA, USA) using a semi-dry trans-blot apparatus. The membranes were blocked for 1 h in 10% nonfat milk in Tris-buffered saline and Tween 20 (TBST) buffer at 37°C and then incubated with specific antibodies overnight at 4°C. After washing three times with TBST, the membranes were incubated with HRP-conjugated secondary antibodies for 1 h at room temperature. Finally, bands were detected using an ECL western blotting detection kit, and the signals were analyzed via densitometry using a GS-710 Imaging Densitometer (Bio-Rad, Hercules, CA, USA). The bands were analyzed by scanning and normalized to β-actin or GAPDH. All data reflect the results of three independent experiments.

### Statistical analysis

Statistical analysis was performed with SPSS 18.0. Unpaired Student’s t-tests were used for the statistical analysis. Measurements are presented as the mean ± SD. Each experiment was repeated at least three times, and P < 0.05 was considered significant.

## SUPPLEMENTARY MATERIALS FIGURE AND TABLE



## Abbreviations

AS: atherosclerosis
CTRP3: C1q/tumor necrosis factor-related protein-3
TLR4-NF-κB: Toll-like receptor 4 (TLR4)-nuclear factor-κB
PPARγ-LXRα-ABCA1/ABCG1: peroxisome proliferative activated receptor γ (PPARγ)-liver-X-receptor (LXR)-ATP binding cassette transporter A1 (ABCA1)/ATP binding cassette transporter G1 (ABCG1)
LPS: lipopolysaccharide
PDTC: pyrrolidine dithiocarbamate
PMA: phorbol-12-myristate-13-acetate
ox-LDL: oxidized LDL
CE:TC: cholesteryl ester:total cholesterol
