# Supplementary material for: C1q/Tumor necrosis factor-related protein-3 protects macrophages against LPS-induced lipid accumulation, inflammation and phenotype transition via PPARγ and TLR4-mediated pathways
Source: Oncotarget. 2017 Jul 28;8(47):82541–57. doi: 10.18632/oncotarget.19657 (PMC5669909; doi:10.18632/oncotarget.19657)
Supplement: Supplementary file 1 [file oncotarget-08-82541-s001.pdf]

C1q/Tumor necrosis factor-related protein-3 protects macrophages against LPS-induced lipid accumulation, inflammation and phenotype transition via PPARγ and TLR4-mediated pathways

SUPPLEMENTARY MATERIALS

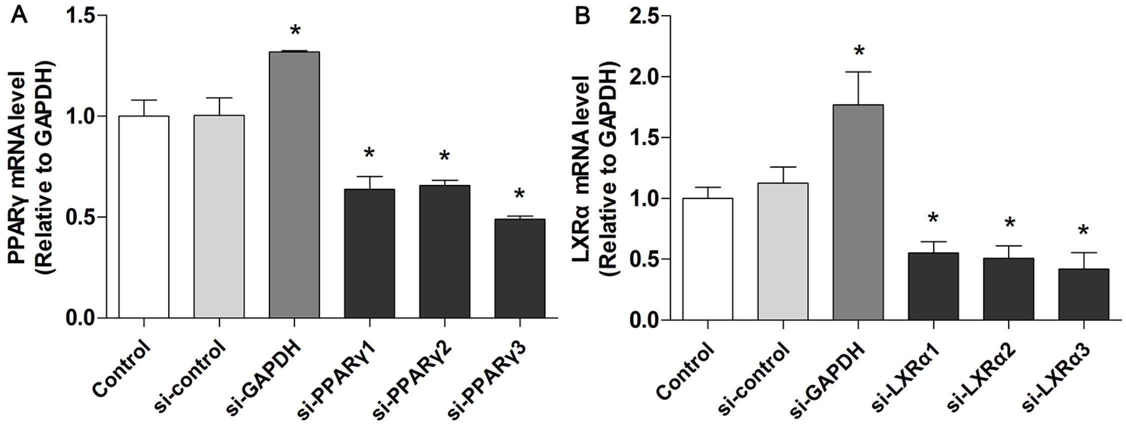

Supplementary Figure 1: Effectiveness of siRNAs verified by RT-PCR. After co-culture with PMA for 48 h, THP-1 cells (1×10^6 cells/mL/well) were transfected with si-control, si-GAPDH or three types of si-PPARγ and si-LXRα; the control group was treated with PBS. After 48 h, mRNA levels were measured by quantitative RT-PCR, with the expression level of PPARγ and LXRα normalized to GAPDH. Data were calculated using the ΔΔCT method, and the results are represented as the mean± SD (*: compared to the control group, $P < 0.05$).

Supplementary Table 1: Primer sequences for qRT-PCR procedures

Category	Sequence
Hum INOS F	5'-CCAGCCTCAAGTCTTATTTCCTC-3'
Hum INOS R	5'-CACTCAGCAGCAAGTTCCATC-3'
Hum IL6 F	5'-AGCCACTCACCTCTTCAGAAC-3'
Hum IL6 R	5'-GCAAGTCTCCTCATTGAATCCAG-3'
Hum TNFα F	5'-GTCTGGGCAGGTCTACTTTGG-2'
Hum TNFα R	5'-GAGGTTGAGGGTGTCTGAAGG-2'
Hum IL-1β F	5'-TGGCTTATTACAGTGGCAATGAG-3'
Hum IL-1β R	5'-GTAGTGGTGGTCGGAGATTCG-3'
Hum CD163 F	5'-CATTATGTCCTTCAGAGCAAGTG-3'
Hum CD163 R	5'-AGCGACCTCCTCCATTTACC-3'
Hum Arginase1 F	5'-CAAGGTGGCAGAAGTCAAGAAG-3'
Hum Arginase1 R	5'-GTGGTTGTCAGTGGAGTGTTG-3'
Hum MR F	5'-GCCAGCGACATAACAGTAGTATC-3'
Hum MR R	5'-AGACCAGATTCCCTCCAAAGC-3'
Hum FIZZ1 F	5'-ACAGTCCCTCTCCTATAAGCAAG-3'
Hum FIZZ1 R	5'-ACCACAGCCATAGCCACAAG-3'
Hum β-actin F	5'- TCGTGCGTGACATTAAGGAGAAG-3'
Hum β-actin R	5'- GTTGAAGGTAGTTTCGTGGATGC -3'
Hum PPARγ F	5'-AAGACATTCCATTCACAAGAACAG-3'
Hum PPARγ R	5'-TTATCTCCACAGACACGACATTC-3'
Hum LXRα F	5'-TTGCCTTGCTCATTGCTATCAG-3'
Hum LXRα R	5'-ATCCGTGGGAACATCAGTCG-3'
Hum GAPDH F	5'-CCACTCCTCCACCTTTGAC-3'
Hum GAPDH R	5'-ACCCTGTTGCTGTAGCCA-3'
